# Lineage-Specific Chiral Biases of Human Embryonic Stem Cells during Differentiation

**DOI:** 10.1155/2018/1848605

**Published:** 2018-12-02

**Authors:** Kathryn E. Worley, Amanda S. Chin, Leo Q. Wan

**Affiliations:** ^1^Department of Biomedical Engineering, Rensselaer Polytechnic Institute, 110 8th Street, Troy NY 12180, USA; ^2^Department of Biological Sciences, Rensselaer Polytechnic Institute, 110 8th Street, Troy NY 12180, USA; ^3^Center for Biotechnology & Interdisciplinary Studies, Rensselaer Polytechnic Institute, 110 8th Street, Troy NY 12180, USA; ^4^Center for Modeling, Simulation and Imaging in Medicine, Rensselaer Polytechnic Institute, 110 8th Street, Troy NY 12180, USA

## Abstract

Left-right symmetry breaking is a complex developmental process and an important part of embryonic axis development. As of yet, the biophysical mechanism behind LR asymmetry establishment remains elusive for the overall asymmetry of embryos as well as for the organ-specific asymmetry. Here, we demonstrate that inherent cellular chirality is observable in the cells of early embryonic stages using a 3D Matrigel bilayer system. Differentiation of human embryonic stem cells to three lineages corresponding to heart, intestine, and neural tissues demonstrates phenotype-specific inherent chiral biases, complementing the current knowledge regarding organ development. The existence of inherent cellular chirality early in development and its correlation with organ asymmetry implicate cell chirality as a possible regulator in LR symmetry breaking.

## 1. Introduction

Left-right (LR) symmetry breaking events are an important aspect of embryogenesis. They are required for the correct placement and orientation of various organs and organ systems. Without them, it can lead to malformations and functional disorders [1]. Common malformations include congenital heart defects, such as transposition of the great arteries, double outlet right ventricle and atrioventricular septal defects [1], and abnormalities in midgut rotation which can lead to abdominal pain and intestinal obstructions [2]. A thorough grasp of LR axis development is crucial to a more complete understanding of these heath issues, so that treatment and prevention can be improved. In particular, the development of *in vitro* platforms for studying LR symmetry breaking, as focused in this study, is of great scientific and clinical importance.

Research into LR symmetry breaking has resulted in various theories based on current evidence in the field. Primary cilia theory is one of the most prevalent theories, depicting how asymmetric flow, generated by beating cilia, and resultant nodal signalling work together in establishing LR patterning [3]. Evidence for this theory can be found in model organisms such as mouse and zebrafish and is thought to be relevant to human development due to disruption of LR asymmetry in disease states such as primary ciliary dyskinesia [3]. In contrast, the intracellular model suggests that LR symmetry breaking occurs early in development, within the first few embryonic cleavages, and depends on cytoskeletal chirality [4]. This model is based on evidence implicating cytoskeletal and motor protein in regulating LR asymmetry in invertebrates such as *C. elegans* and *Drosophila*, as well as in the vertebrate *Xenopu*s [5]. A unifying model has also been proposed, combining research on the cytoskeleton and ion channels (chick, *C. elegans*, and *Xenopus*) in early development with research done at later stages, such as cilia flow [4]. This model proposes that early asymmetries initiate LR symmetry breaking while later events act to maintain or amplify these asymmetries [4]. While some mechanisms are likely to be conserved amongst species, further studies will be needed to determine the extent to which similarities exist. As of yet, there are no decisive models able to link early symmetry breaking events all the way to asymmetric organ formation. This is especially important to consider when we look into research on the development of certain organs.

The heart, gut, and brain display marked asymmetries in adults, which originate early in development. All three of these organs start off as straight tubes situated along the midline [6–8]. As the development of the heart and gut progresses, they ultimately undergo asymmetric rotations where the tubes experience a clockwise (CW) rotation [6] and several counterclockwise (CCW) rotations [7], respectively. While there exists a CCW cerebral torqueing of the human brain [9], a corresponding rotation of the neural tube has not been visualized and its mechanism of development is currently unknown.

The nodal signalling pathway has been implicated in the establishment of LR asymmetry in heart, gut, and brain development [3], but recent literature indicates that there exists a tissue intrinsic component [10–12]. In zebrafish, a nodal-independent mechanism behind heart looping was discovered, which was dependent on actin polymerization [12]. In *Drosophila* hindgut, chiral cell shape was found to be sufficient to induce asymmetric looping [10]. Additionally, intrinsic planar cell polarity, and thus looping direction, was found to depend on DE-cadherin [10] as well as on Myosin31DF [11]. These works indicate a potentially important role for inherent cellular chirality in the establishment of organ LR asymmetry. The dependence on the actin cytoskeleton is similar to the findings of previous studies which examined LR asymmetry *in vitro* at the cellular level, also termed cell chirality [13]. Additionally, this actin-dependent chirality has been observed at the single-cell level in the embryos of *C. elegans* [14, 15] and in *in vitro* culture on circular micropatterns [16].

The mechanisms behind the establishment of LR asymmetry during embryonic development are currently being debated, with existing work in the field relying primarily on genetic animal models, which are often challenging and may not always recapitulate human development. Here, we propose to use human embryonic stem cell- (hESC-) based *in vitro* models to simulate organ-specific LR symmetry breaking. Using a 3D bilayer Matrigel system and the differentiation of hESCs, lineage-dependent chiral biases in 3D cell rotation are examined at various stages of differentiation into heart, gut, and brain tissues. We demonstrate the potential for inherent cellular chirality to play an important role in the establishment of organ LR asymmetry.

## 2. Materials and Methods

### 2.1. Human Embryonic Stem Cell Culture

The Rockefeller University Embryonic Stem Cell line, RUES2, was utilized in all experiments. These cells were obtained through the Rensselaer Center for Stem Cell Research (RCSCR), a core facility, located in the Center for Biotechnology and Interdisciplinary Studies at Rensselaer Polytechnic Institute. The RUES2 cell line was maintained on Matrigel- (Corning) coated tissue culture plastic in mouse embryonic fibroblast-conditioned media (MEF-CM). Cells were maintained through enzymatic passaging. Briefly, cells were treated with 1 U/mL of dispase (STEMCELL Technologies) for seven minutes and rinsed, and a cell lifter was used to remove cells from the surface. The pluripotency of these hESCs was evaluated through Oct4 (Abcam), SOX2 (Cell Sig. Tech.), and Nanog (Fisher Sci.) immunostaining. Cells are tested monthly for mycoplasma.

### 2.2. Cardiomyocyte Differentiation

Cardiomyocyte differentiation is achieved using a slightly modified version of the protocol described in Lian et al. [17]. The hESCs are single-cell-passaged, seeded onto Matrigel-coated tissue culture plastic, and initially cultured in MEF-CM. These cells are allowed to reach 80–90% confluence over the course of 3–4 days prior to differentiation. Differentiation is induced using CHIR99021 (R&D Systems), a selective inhibitor of glycogen synthase kinase 3, IWP2 (Tocris Biosciences), a WNT signalling inhibitor, and various media (see Supplementary Figure S1 for details). We focus on day 1 mesoendoderm, day 5 cardiac mesoderm, day 8 cardiac progenitors, and day 15 cardiomyocytes. Day 1 mesoendoderm and day 5 cardiac mesoderm utilize the RPMI/B-27 minus insulin media as their base culture media for downstream experiments. Day 8 cardiac progenitors and day 15 cardiomyocytes utilize the RPMI/B-27 media, which contains insulin.

### 2.3. Intestinal Differentiation

A protocol developed to obtain human intestinal tissue from human embryonic stem cells was utilized with slight modifications [18]. This protocol goes through several phases beginning with hESC colonies through to the creation of intestinal organoids. For our purposes, we chose an earlier endpoint, mid/hindgut, due to a greater uniformity in cell type at that stage. We begin mid/hindgut differentiation by growing hESC colonies in MEF-CM on Matrigel-coated dishes to 85–90% confluence. The cells typically reach the appropriate confluency in 2–4 days, and then the differentiation was induced. Differentiation involves two stages: the first uses activin A (PeproTech) to induce endoderm and the second uses a combination of WNT3A (R&D Systems) and FGF4 (PeproTech) to induce mid/hindgut formation (Supplemental Figure S1). The focus for this study is on the day 3 endoderm along with the day 7 mid/hindgut monolayer. Both the endoderm and mid/hindgut cell lines are cultured in RPMI 1640 supplemented with 2% dFBS (HyClone) for all downstream experiments.

### 2.4. Neuroepithelium Differentiation

Neuroepithelium differentiation to primarily central nervous system epithelia requires the single-cell passaging of hESCs and the culturing of these cells to nearly 100% confluence on Matrigel-coated plates prior to beginning neural induction [19, 20]. Upon reaching 100% confluence, the MEF-CM medium is replaced with N2/B27 Neural Induction medium supplemented with the small molecules SB431542 (Tocris Biosciences) and LDN193189 (Sigma). N2/B27 Neural Induction medium consists of DMEM/F12 (Gibco) as the base, N2 supplement (Invitrogen), B27 supplement minus vitamin A (Invitrogen), L-glutamine (2 mM), penicillin/streptomycin, beta-mercaptoethanol (Sigma), and MEM nonessential amino acids (Sigma). The supplemented small molecules inhibit BMP and TGF-*β* signalling which is paramount to successful neural induction. The medium is changed daily with a daily addition of the small molecules to final concentrations of 10 *μ*M and 200 nM, respectively (Supplemental Figure S1). The focus for this study is the day 5 intermediate stage and the day 10 neuroepithelium. Further culturing of these cells takes place in the N2/B27 Neural Induction media without factors.

### 2.5. Endpoint Cell Line Culture

Three cell lines (HL-1, FHs 74 Int, and NE-4C) are used for endpoint comparison for each differentiation. All cell lines were cultured and passaged according to manufacturers' guidelines. In short, the mouse cardiac muscle cell line, HL-1 (EMD Millipore), was cultured in Claycomb Medium (Sigma) supplemented with 10% fetal bovine serum, penicillin/streptomycin (100 *μ*g/mL), norepinephrine (0.1 mM), and L-glutamine (2 mM). Flasks were precoated with a gelatin/fibronectin solution for at least an hour at 37°C prior to seeding, and cells were fed with new media daily. The small intestinal cell line, FHs 74 Int (ATCC® CCL-241™), was cultured in Hybri-Care Medium ATCC 46-X (ATCC) prepared according to the manufacturer's specifications and supplemented with 10% fetal bovine serum and epidermal growth factor (EGF, 30 ng/mL). The mouse neuroectoderm cell line, NE-4C (ATCC® CRL-2925™), was cultured in ATCC-formulated Eagle's minimum essential medium (ATCC) supplemented with L-glutamine (2 mM) and 10% fetal bovine serum. In order to induce PAX6 expression, the NE-4C cells were treated with 1 *μ*M of retinoic acid (RA) for 2 days referred to as NE-4C RA in the results [21].

### 2.6. Immunostaining

The protocols for immunostaining are based on the manufacturer's guidelines. In general, the samples are fixed in 4% paraformaldehyde for 10 minutes and then washed with PBS three times for five minutes each. A solution of 0.3% Triton-X 100 in PBS (PBST) is used in a permeabilization step to open up the cell membrane and allow for the antibodies to enter. The sample is blocked using a serum related to the animal the secondary antibody (goat or donkey) is from and then incubated in the primary antibody solution containing PBST and 1% bovine serum albumin (BSA) for either one hour at room temperature or overnight at 4°C. Three PBS wash steps are performed for five minutes each, and the sample is incubated in a secondary antibody solution containing PBST and 1% BSA for one hour at room temperature. The sample is then washed three times in PBS for five minutes each and mounted using fluorescent mounting media containing DAPI. The following markers were used to determine differentiation efficiency: brachyury (R&D Systems) for mesoendoderm, Nkx2.5 (Abcam) for cardiac mesoderm, Nkx2.5 and Isl1 (Developmental Studies Hybridoma Bank) for cardiac progenitors, cTnT (Thermo Fisher Scientific) for cardiomyocytes, double staining of FOXA2 (GeneTex) and SOX17 (GeneTex) for endoderm, CDX2 (GeneTex) for mid/hindgut, Otx2 (R&D Systems) for day 5 neural induction, and PAX6 (GeneTex) for neuroepithelium [17–20]. Antibody catalogue numbers and dilutions can be found in Supplemental Table S1.

### 2.7. Three-Dimensional Matrigel Bilayer System

A 3D bilayer Matrigel system consisting of a 100% bottom layer and 2% top layer was used in all experiments. This bilayer system creates a flat interface for imaging and a hydrogel gradient that allows for consistent orientation of the cells through a defined *z*-axis. Prior to experiments, all hESC-derived lineages were treated for one hour with the ROCK inhibitor, Y27632 (Stemgent), to facilitate cell survival. The bottom of an eight-chambered glass slide (ibidi) is first coated with a base layer of 100% Matrigel and allowed to gel for 20 minutes at 37°C [22]. The cells are then single-cell-passaged using trypsin (Gibco) and are seeded at a seeding density of 10,000 cells per cm^2^ onto the base Matrigel layer and allowed to attach for 15–30 minutes. After attachment, the media is removed and replaced with cold media containing 2% Matrigel. This Matrigel-media solution is then incubated at 37°C and creates the top layer [23]. The cells are now encased in a 3D Matrigel bilayer environment [22], and time-lapses are typically run for 2–4 hours at 1–5-minute intervals. Imaging is done with a Keyence BZ-X700 microscope with incubation setup, and images are gathered at a 10X magnification.

### 2.8. 3D Chirality Assay Analysis

**Figure 1:**
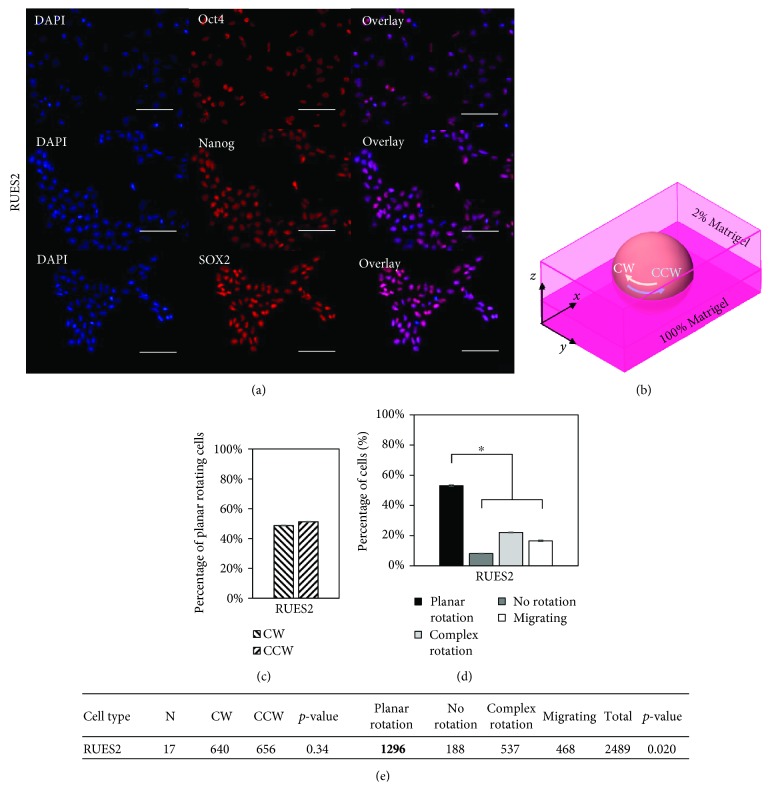
Human embryonic stem cell line, RUES2, displays no chiral bias. (a) Representative images of RUES2 cells expressing the nuclear marker DAPI (blue) and pluripotency markers Oct4, Nanog, and SOX2 (red). Over 95% of RUES2 cells exhibit Oct4, Nanog, and SOX2 expression. (b) A schematic showing planar rotation in the *x*-*y* plane about the *z*-axis. (c) RUES2 cells do not show a definitive chiral bias in rotation, displaying an almost 50 : 50 ratio of CW to CCW rotating cells. (d) A statistically significant number of cells rotated in plane compared to the other categories. (e) Quantitative data showing the number of cells in each category along with the number of replicate experiments (*N*). The *p* values indicate comparisons between CW and CCW and between planar rotation and the other three categories. Scale bars: 100 *μ*m; ^∗^ *p* < 0.05.

Cells were analysed visually by quickly scrolling through the phase-contrast time-lapse videos in ImageJ and observing the motion of individual cells. Rotating cells can be classified into three categories: no rotation, complex rotation, and in-plane rotation. The bilayer Matrigel system is thought to create a mechanical gradient which allows the cells to differentiate between top and bottom. This layered system causes the majority of cells to rotate around the *z*-axis, which when imaging would be the axis coming out of the image (Figure 1(b)). In-plane rotation is classified as rotation purely around the *z*-axis and is broken down into clockwise (CW) and counterclockwise (CCW) rotations. Complex rotation is rotation that is not purely around the *z*-axis or rotation that switches between CW and CCW. Depending on the cell type, we also see migration of the cells through the gel construct; these cells are categorized by their spread morphology, and thus we create a further classification, migrating. A classification of planar rotation was assigned if a persistent rotational movement, characterized by a flow of features either CW or CCW, was observed visually for the entire time frame of the video. The other categories were likewise assigned based on observations of time-lapse videos. Representative videos of each classification are provided in the Supplementary Materials (Supp. Videos S1–10). For tracked videos, phase-contrast images were tracked in Image J using MTrackJ at 10-minute intervals. A comprehensive training video has also been included which details the classifications and ImageJ analysis (Supp.  Video 11).

### 2.9. Statistical Analysis

Rank tests were used to determine whether there is a significant direction bias between CW and CCW rotating cells. The binomial cumulative distribution function (binocdf) in MATLAB was used to evaluate if the ratio of CW or CCW rotating cells significantly differed from 50%. A similar analysis was used to evaluate the percentage of planar rotating cells and a combination of the other three categories (no rotation, complex rotation, and migrating). Statistical significance was determined at a confidence level *α* = 0.05.

## 3. Results

A phenotype-dependent inherent chiral bias in migration and rotation has been previously established [22, 24]. The importance of this dependence has yet to be determined, although it likely plays a role in the formation of organ left-right asymmetry during development. We utilize a 3D chirality assay to observe the biased rotation of hESCs and derived lineages to determine the stages where chiral bias becomes apparent and if this bias corresponds to biases in established cell lines of a similar phenotype.

### 3.1. hESCs Display No Chiral Bias in Rotation

The hESC line, RUES2, displays no chiral bias in the 3D bilayer system, indicated by the nearly 50 : 50 ratio of CW to CCW planar rotating cells (Figures 1(c) and 1(e)). However, a significant number of the cells observed rotated within the *x*-*y* plane about the *z*-axis (Figures 1(b)–1(e)). The pluripotency of the hESCs was determined through Oct4 expression (Figure 1(a)) and found to be above 95% (Table S2), which is typical of hESC populations.

### 3.2. Cardiac Mesoderm Exhibits a CW Rotational Bias

**Figure 2:**
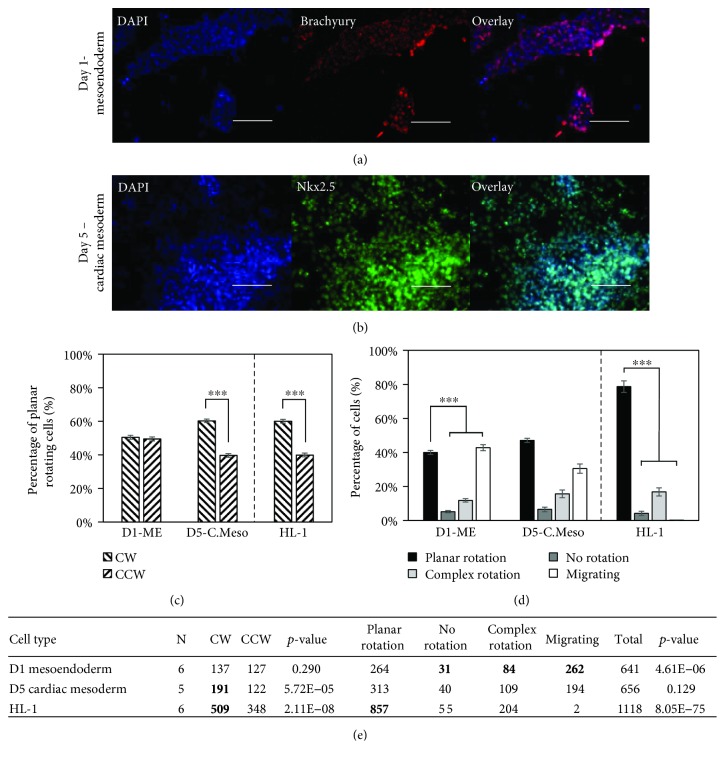
Cardiac mesoderm cells display dominant CW biased rotation similar to a mouse cardiac cell line, HL-1. (a) Day 1 mesoendoderm cells expressing brachyury. (b) Day 5 cardiac mesoderm cells expressing Nkx2.5 (c) Mesoendoderm (D1-ME) shows no rotational bias, and cardiac mesoderm (D5-C.Meso) displays a CW bias along with the mouse cardiac muscle cell line, HL-1. (d) Mesoendoderm has more migrating, no-rotation, and complex rotating cells than planar rotating cells. Cardiac mesoderm shows no difference between the two sets of groups while HL-1 cells are primarily planar rotating. (e) Quantitative data showing the number of cells in each category along with the number of replicate experiments (*N*). The *p* values indicate comparisons between CW and CCW and between planar rotation and the other three categories. Scale bars: 100 *μ*m; ^∗∗∗^ *p* < 0.001.

The RUES2 cell line was successfully differentiated to a cardiac lineage, illustrated by typical cardiac marker staining for each stage (Figures 2(a) and 2(b); Supplemental Figure S2a-b; Supplemental Table S2). The day 5 cardiac mesoderm stage of cardiac differentiation shows a significant CW bias in rotation which is comparable to that seen for the mouse cardiac muscle cell line HL-1 (Figures 2(c) and 2(e)). The day 1 mesoendoderm shows no bias in rotation (Figures 2(c) and 2(e)); similar to the hESCs (Figures 1(c) and 1(e)), day 8 cardiac progenitors and day 15 cardiomyocytes both showed a slight CW bias, but it was not statistically significant (Supplemental Figure S2c and S2e). The 3D bilayer system was able to evaluate two of the four cardiac differentiation stages and the cardiac cell line effectively as demonstrated by high numbers of planar rotating cells for the mesoendoderm and cardiac mesoderm stages and HL-1 cells (Figures 2(d) and 2(e)). Unfortunately, the highly migratory nature of the cardiac progenitor cells and cardiomyocytes made the evaluation of any chiral bias in rotation difficult due to a low number of planar rotating cells (Supplemental Figure S2d and S2e).

### 3.3. Intestinal Cell Lineages Demonstrate CCW Rotational Bias

**Figure 3:**
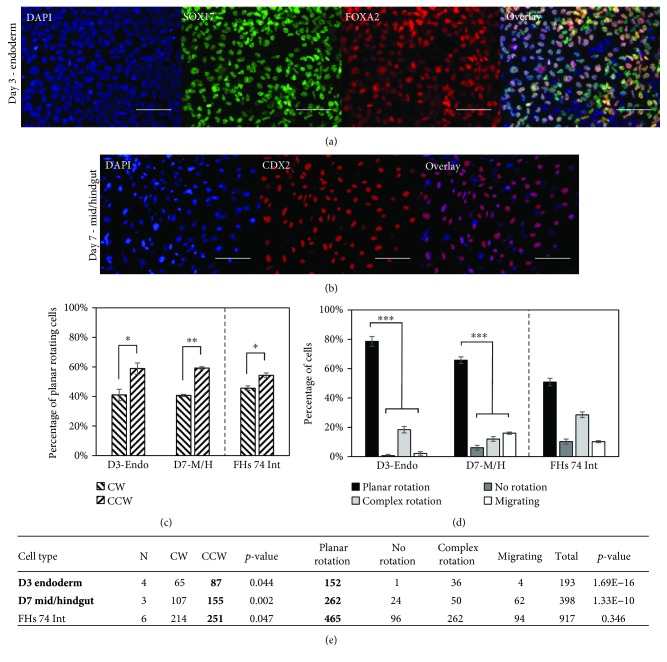
Human ESCs differentiated to an intestinal lineage display dominant CCW biased rotation, which is comparable to an intestinal cell line. (a) Representative images of cells expressing the SOX17 and FOXA2 markers indicating endoderm differentiation. (b) Representative images of cells expressing an CDX2 marker indicating mid/hindgut differentiation. (c) Endoderm (D3-Endo), mid/hindgut (D7-M/H), and the primary small intestinal cell line (FHs 74 Int) all display statistically significant CCW rotational bias. (d) Endoderm and mid/hindgut show significantly more planar rotating cells than other categories; however, the FHs 74 Int cell line does not. (e) Quantitative data showing the number of cells in each category along with the number of replicate experiments (*N*). The *p* values indicate comparisons between CW and CCW and between planar rotation and the other three categories. Scale bars: 100 *μ*m; ^∗^ *p* < 0.05; ^∗∗∗^ *p* < 0.01.

Differentiation of RUES2 cells to an intestinal lineage resulted in an efficiency of approximately 60% for both endoderm and mid/hindgut differentiations (Figures 3(a) and 3(b), Table S2). This was a lower efficiency than expected; therefore, we performed additional staining of cells after quantification in the 3D system and found the samples to contain over 85% of the differentiation marker (Supplemental Figure S3a). We found that cells expressing endoderm or mid/hindgut differentiation markers exhibited a statistically significant increase in CCW biased cells. This CCW bias can be seen as early as the establishment of day 3 endoderm and becomes even more apparent in day 7 mid/hindgut (Figures 3(c) and 3(e)). Further testing of endoderm using a more robust differentiation protocol showed similar results (Supplemental Figure S4). These results correspond to the early gestational (3–4-month) human small intestinal cell line, FHs 74 Int (Figures 3(c) and 3(e)). Additionally, cells rotating within the *x*-*y* plane were significantly more likely than the other categories, except for the FHs 74 Int cell line which showed no difference (Figures 3(d) and 3(e)). Cells that did not express the markers of interest were also quantified and found to exhibit CCW bias for the endoderm sample and no bias for the mid-hindgut sample (Supplemental Figure S3b, S3c, and S3d).

### 3.4. Neural Ectoderm Shows a CCW Rotational Bias While Neuroepithelium Does Not

**Figure 4:**
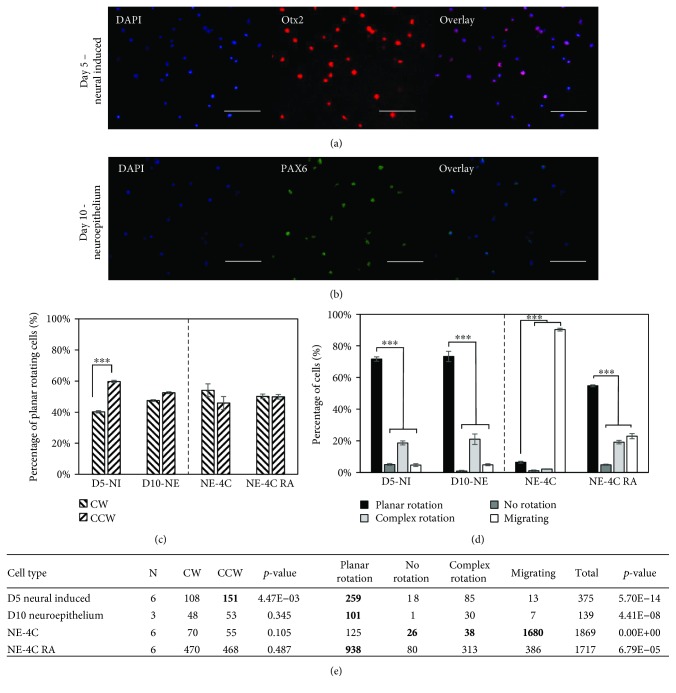
The intermediate neural differentiation stage displays a CCW rotation bias, while neuroepithelium does not. (a) Day 5 neural-induced cells expressing Otx2, a neural ectoderm marker. (b) Day 10 neuroepithelium expressing PAX6, a neural lineage marker. (c) Neural-induced cells (D5-NI) show a statistically significant CCW rotational bias while neuroepithelium (D10-NE) and the neuroepithelial cell line NE-4C, both treated with retinoic acid (NE-4C RA) and untreated (NE-4C), do not. (d) All groups show significantly more planar rotating cells than other categories, except the NE-4C group which is mostly migratory. (e) Quantitative data showing the total number of cells in each category along with the number of replicate experiments (*N*). The *p* values indicate comparisons between CW and CCW and between planar rotation and the other three categories. Scale bars: 100 *μ*m; ^∗∗∗^ *p* < 0.001.

The efficiency of neuroepithelial differentiation was as expected indicated by the expression of Otx2 and PAX6 (Figures 4(a) and 4(b); Table S2). At the day 5 stage of neural induction, the cells exhibited a statistically significant CCW bias in rotation, but this bias was ultimately lost at day 10 (Figures 4(c) and 4(e)). A comparison to the mouse neuroectodermal cell line NE-4C showed no rotational bias; however, it showed a surprising amount of migratory cells (Figures 4(c)–4(e)). These cells do not typically express PAX6 unless treated with retinoic acid (RA) [21]; treatment of the NE-4C cells with RA resulted in a similar outcome to that of day 10 neuroepithelial induction (Figures 4(c)–4(e)). Ultimately, neural induction resulted in very little change in the percentage of planar rotating cells (Figures 4(d) and 4(e)) but did show a transient CCW chiral bias over the course of differentiation.

## 4. Discussion

In this study, we show for the first time that inherent chirality of embryonic stem cells and derived lineages can be evaluated using a 3D *in vitro* assay. Assessment of biased rotation using our 3D Matrigel bilayer system demonstrated altered biases at early embryonic time points during hESC differentiation in a lineage-specific manner. Biased cell rotation is seen as a dominant CW bias in day 5 cardiac mesoderm and CCW biases in day 3 endoderm, day 7 mid/hindgut, and day 5 neural induced stages. The chiral biases seen in these stages also correspond to those seen in cell lines of similar types. These findings indicate that cell chirality is cell phenotype-dependent and may contribute to symmetry breaking at particular time points associated with specific cell differentiation during embryonic development.

There is a correlation between *in vitro* cell chirality and organ-specific rotation during embryonic development. The embryonic heart tube is initially straight and bilaterally symmetric across the midline, but it eventually undergoes a consistent CW rotation and dextral bending that ultimately results in a leftward pointing C-shaped concavity [6]. The CW rotation of cardiac mesoderm stage cells corresponds to the CW rotation of the heart tube during development [6]. Inherent cell chirality has previously been shown to be important for the CCW rotation of the *Drosophila* hindgut [10, 11]. The CCW bias demonstrated by the rotation of the day 3 endoderm and day 7 mid/hindgut cells agrees with these results, as well as the known CCW rotation of the midgut in human development [2]. The transient nature of the rotational bias in the neural lineage differentiation was unanticipated. The day 5 neural-induced stage demonstrated a CCW rotation bias which corresponds to CCW cerebral torque [9]. However, how these would be related is not clear. The randomization of this chiral bias at day 10 indicates that perhaps a chiral bias is important only at certain stages or cell types in brain development. All our findings suggest that cell chirality may contribute to organ-specific LR asymmetry through the chiral bias obtained during stem cell lineage-specific differentiation.

There are similarities and inconsistencies in cell chirality of differentiated hESCs and the corresponding endpoint cell lines. The day 10 neuroepithelium was consistent with the mouse neuroectodermal cell line NE-4C after RA treatment. A comparison of the mid/hindgut lineages to a primary small intestinal cell line (FHs 74 Int) was consistent, however not quite as distinctive as the chiral bias in the differentiated cells, and this may be due to the combined nature of the cell line as it was not specific to midgut. As for cardiac lineage differentiation, the day 5 cardiac mesoderm findings and the mouse cardiac muscle cell line, HL-1, have the same chiral biases. However, the HL-1 cells were, surprisingly, found to be primarily planar rotating in contrast to the high migratory nature of the day 8 cardiac progenitors and day 15 cardiomyocytes. A highly migratory nature was expected as cardiac progenitors are seen to migrate during development [25, 26]. It is possible that the HL-1 cells had partially lost their cardiomyocytic phenotype, as observed in their diminishing beating behaviour after multiple passages [27]. In addition, this may be a species-specific response as this cell line was derived from mouse tissue as opposed to human tissue.

The mechanisms behind chiral biases in cell rotation and their alterations during differentiation are still unclear. Differential gene expression in the cell population could be a reason behind some of the categories (CW/CCW/no rotation/complex rotation/migrating), but what those genes are is currently unknown. The undifferentiated hESCs studied here show randomized planar rotation, but this randomness does not appear to be directly associated with pluripotency. The expression of Oct4, SOX2, and Nanog is higher than 95%, and none of the categories have an equally high percentage making the expression of pluripotency markers an unlikely predictor. Human ESCs are known to be heterogeneous in their gene expression [28], and this varying gene expression within the population has been shown to facilitate differentiation of certain subpopulations [29, 30]. Additionally, we see that the differentiations themselves are not 100% efficient, although similar to Oct4 expression in hESCs, the percentages of differentiation markers do not seem to explain any one category for any stage. The current knowledge in the field implicates the actin cytoskeleton and the actin cross-linker *α*-actinin-1 [16] in inherent cellular chirality *in vitro* [16, 24, 31–33] as well as in LR symmetry breaking *in vivo* [14, 15, 34]. Future studies should consider how hESC heterogeneity and differentiation into different lineages impact gene expression related to cytoskeletal dynamics in order to discern the nature of chiral bias. Furthermore, research into how different signalling pathways, especially those known to influence LR asymmetric development, can alter this bias through changes in cytoskeletal dynamics should be explored.

The chiral bias of several early lineage cell types was able to be discerned through the use of a 3D Matrigel bilayer system, but some considerations have to be taken into account in data explanation. Local fluctuations in matrix composition or mechanical properties may be a cause of the various assay categories and certain levels of randomness in the direction of cell rotation. There are a variety of potential reasons why cells may fall outside of the planar rotation category, with some of the most likely discussed here. In the small percentage of the no-rotation category, it is possible that the cells are dying and therefore no longer rotating, or they are perhaps in the process of transitioning to a different category. Complex rotation is likely induced by slight differences in the environment surrounding the cell, causing a lack of defined axes, by cells transitioning to different categories or by bicells of differing chirality interacting. The category of migration likely corresponds to an increase in mesenchymal-like cells especially for groups where the majority of the cells migrate. A variation in cell attachment timing may cause a portion of the cells to begin spreading prior to the addition of the second layer. Seeding density can also cause variability in cell behaviour as well. Cells very close to each other have been seen to start migrating toward each other in the assay, which could increase the incidence of migrating cells or complex behaviour resulting from cells of different chirality interacting. However, seeding density should have a minimal effect on the outcome of the assay since cells in very close proximity were excluded from analysis. Future studies should look into creating a universal 3D chirality assay that is able to quantify rotation for any cell type by utilizing a uniform matrix that cells can attach to, but not necessarily remodel, in order to maximize the number of planar rotating cells.

The evaluation of tissue types corresponding to mesoderm, endoderm, and ectoderm, the three germ layers, illustrates that these early tissues may already display chiral bias that could influence the formation of later organs. This is an important point, since the formation of the germ layers coincides with the development of the node, making cilial flow unlikely to be the cause of inherent cellular chirality [35]. In fact, work done in mouse has demonstrated that node cells themselves undergo a planar polarization which facilitates the direction of cilia rotation [36]. The existence of chiral bias in cells at very early stages of development gives further credence to a unifying model [4] of LR asymmetry. Additionally, work done using zebrafish has also implicated the asymmetric expression of nodal, produced by cilial flow, in amplifying preexisting tissue bias, through changes in actin genes [12]. Our results implicate inherent cellular chirality as a potential contributor to the establishment of LR asymmetry. Future work is necessary to examine how this cellular property may work together with known components of LR symmetry breaking pathways to regulate LR asymmetry in development.

## 5. Conclusions

There are currently many theories involving how LR asymmetry is established. We demonstrate here that inherent cellular chirality can be seen in early tissue types which liken several organs with distinctive LR asymmetry to those of adults. Cell lineages corresponding to the heart, intestine, and brain all revealed phenotype-specific inherent chiral bias. These lineages displayed chiral bias that was comparable to established cell lines of similar phenotype and paralleled current knowledge regarding organ development. Our results give further credence to the idea that inherent cellular chirality plays a role in events leading to developmental LR symmetry breaking.

## Data Availability

All data that support the findings of this study are available within the published manuscript and its supplementary information or from the corresponding author (LQW) on reasonable request.

## Supplementary Materials

Supplementary Materials The supplemental materials contain information detailing the differentiation protocols, the antibody information including catalogue numbers and concentrations used for immunostaining, tables describing differentiation efficiencies for each stage, and the 3D bilayer rotation data corresponding to D8 cardiac progenitors and D15 cardiomyocytes. Videos showing the different categories (CW, CCW, no rotation, complex, and migrating) used for analysis are also included.

## References

[1] Peeters H., Devriendt K. (2006). Human laterality disorders. *European Journal of Medical Genetics*.

[2] Langer J. C. (2017). Intestinal rotation abnormalities and midgut volvulus. *The Surgical Clinics of North America*.

[3] Grimes D. T., Burdine R. D. (2017). Left-right patterning: breaking symmetry to asymmetric morphogenesis. *Trends in Genetics*.

[4] Vandenberg L. N., Levin M. (2013). A unified model for left-right asymmetry? Comparison and synthesis of molecular models of embryonic laterality. *Developmental Biology*.

[5] Levin M., Palmer A. R. (2007). Left-right patterning from the inside out: widespread evidence for intracellular control. *BioEssays*.

[6] Moorman A., Webb S., Brown N. A., Lamers W., Anderson R. H. (2003). Development of the heart: (1) formation of the cardiac chambers and arterial trunks. *Heart*.

[7] de Santa Barbara P., van den Brink G. R., Roberts D. J. (2003). Development and differentiation of the intestinal epithelium. *Cellular and Molecular Life Sciences*.

[8] Stiles J., Jernigan T. L. (2010). The basics of brain development. *Neuropsychology Review*.

[9] Toga A. W., Thompson P. M. (2003). Mapping brain asymmetry. *Nature Reviews. Neuroscience*.

[10] Taniguchi K., Maeda R., Ando T. (2011). Chirality in planar cell shape contributes to left-right asymmetric epithelial morphogenesis. *Science*.

[11] Hatori R., Ando T., Sasamura T. (2014). Left-right asymmetry is formed in individual cells by intrinsic cell chirality. *Mechanisms of Development*.

[12] Noel E. S., Verhoeven M., Lagendijk A. K. (2013). A nodal-independent and tissue-intrinsic mechanism controls heart-looping chirality. *Nature Communications*.

[13] Wan L. Q., Ronaldson K., Guirguis M., Vunjak-Novakovic G. (2013). Micropatterning of cells reveals chiral morphogenesis. *Stem Cell Research & Therapy*.

[14] Schonegg S., Hyman A. A., Wood W. B. (2014). Timing and mechanism of the initial cue establishing handed left-right asymmetry in Caenorhabditis elegans embryos. *Genesis*.

[15] Naganathan S. R., Furthauer S., Nishikawa M., Julicher F., Grill S. W. (2014). Active torque generation by the actomyosin cell cortex drives left-right symmetry breaking. *eLife*.

[16] Tee Y. H., Shemesh T., Thiagarajan V. (2015). Cellular chirality arising from the self-organization of the actin cytoskeleton. *Nature Cell Biology*.

[17] Lian X., Zhang J., Azarin S. M. (2013). Directed cardiomyocyte differentiation from human pluripotent stem cells by modulating Wnt/*β*-catenin signaling under fully defined conditions. *Nature Protocols*.

[18] McCracken K. W., Howell J. C., Wells J. M., Spence J. R. (2011). Generating human intestinal tissue from pluripotent stem cells *in vitro*. *Nature Protocols*.

[19] Chambers S. M., Fasano C. A., Papapetrou E. P., Tomishima M., Sadelain M., Studer L. (2009). Highly efficient neural conversion of human ES and iPS cells by dual inhibition of SMAD signaling. *Nature Biotechnology*.

[20] Ozair M. Z., Noggle S., Warmflash A., Krzyspiak J. E., Brivanlou A. H. (2013). SMAD7 directly converts human embryonic stem cells to telencephalic fate by a default mechanism. *Stem Cells*.

[21] Varga B. V., Hadinger N., Gocza E. (2008). Generation of diverse neuronal subtypes in cloned populations of stem-like cells. *BMC Developmental Biology*.

[22] Chin A. S., Worley K. E., Wan L. Q. Collective chiral rotation of epithelial microtissues within a three-dimensional matrigel system. http://archive.sb3c.org/2015-proceedings/.

[23] Debnath J., Muthuswamy S. K., Brugge J. S. (2003). Morphogenesis and oncogenesis of MCF-10A mammary epithelial acini grown in three-dimensional basement membrane cultures. *Methods*.

[24] Wan L. Q., Ronaldson K., Park M. (2011). Micropatterned mammalian cells exhibit phenotype-specific left-right asymmetry. *Proceedings of the National Academy of Sciences of the United States of America*.

[25] Munsterberg A., Yue Q. (2008). Cardiac progenitor migration and specification: the dual function of Wnts. *Cell Adhesion & Migration*.

[26] Smith K. A., Chocron S., von der Hardt S. (2008). Rotation and asymmetric development of the zebrafish heart requires directed migration of cardiac progenitor cells. *Developmental Cell*.

[27] Claycomb W. C., Lanson N. A., Stallworth B. S. (1998). HL-1 cells: a cardiac muscle cell line that contracts and retains phenotypic characteristics of the adult cardiomyocyte. *Proceedings of the National Academy of Sciences of the United States of America*.

[28] Graf T., Stadtfeld M. (2008). Heterogeneity of embryonic and adult stem cells. *Cell Stem Cell*.

[29] Gundry R. L., Burridge P. W., Boheler K. R. (2011). Pluripotent stem cell heterogeneity and the evolving role of proteomic technologies in stem cell biology. *Proteomics*.

[30] Drukker M., Tang C., Ardehali R. (2012). Isolation of primitive endoderm, mesoderm, vascular endothelial and trophoblast progenitors from human pluripotent stem cells. *Nature Biotechnology*.

[31] Yamanaka H., Kondo S. (2015). Rotating pigment cells exhibit an intrinsic chirality. *Genes to Cells*.

[32] Liu W., Bao Y., Lam M. L. (2016). Nanowire magnetoscope reveals a cellular torque with left-right bias. *ACS Nano*.

[33] Tamada A., Igarashi M. (2017). Revealing chiral cell motility by 3D Riesz transform-differential interference contrast microscopy and computational kinematic analysis. *Nature Communications*.

[34] Naganathan S. R., Middelkoop T. C., Furthauer S., Grill S. W. (2016). Actomyosin-driven left-right asymmetry: from molecular torques to chiral self organization. *Current Opinion in Cell Biology*.

[35] Lee J. D., Anderson K. V. (2008). Morphogenesis of the node and notochord: the cellular basis for the establishment and maintenance of left-right asymmetry in the mouse. *Developmental Dynamics*.

[36] Hashimoto M., Shinohara K., Wang J. (2010). Planar polarization of node cells determines the rotational axis of node cilia. *Nature Cell Biology*.

