# Supplementary material for: Lineage-Specific Chiral Biases of Human Embryonic Stem Cells during Differentiation
Source: Stem Cells Int. 2018 Dec 2;2018:1848605. doi: 10.1155/2018/1848605 (PMC6304839; doi:10.1155/2018/1848605)
Supplement: Supplementary Materials — The supplemental materials contain information detailing the differentiation protocols, the antibody information including catalogue numbers and concentrations used for immunostaining, tables describing differentiation efficiencies for each stage, and the 3D bilayer rotation data corresponding to D8 cardiac progenitors and D15 cardiomyocytes. Videos showing the different categories (CW, CCW, no rotation, complex, and migrating) used for analysis are also included. [file 1848605.f1.zip › Supplemental/Supplemental_Differentiation_Manuscript_09192018.pdf]

Supplementary Materials

Table S1. Antibodies used for characterization of hESC pluripotency and differentiation efficiencies.

Antibody	Cell Stage	Manufacturer	Catalog #	Concentration
Oct4	hESC	Abcam	ab184665	1:100
SOX2	hESC	Cell Signaling Technology	3579S	1:400
Nanog	hESC	Thermo Fisher	MA1-017	1:200
SOX17	definitive endoderm	GeneTex	GTX83582	1:100
FOXA2	definitive endoderm	GeneTex	GTX62638	1:250
CDX2	mid/hindgut	GeneTex	GTX113160	1:200
Brachyury	mesoendoderm	R&D Systems	AF2085	1:100
Nkx2.5	cardiac mesoderm & cardiac progenitors	Santa Cruz Biotechnology	sc-376565	1:100
Isl1	cardiac progenitors	Developmental Studies Hybridoma Bank	39.4D5	1:20
cTnt	cardiomyocytes	Thermo Fisher	MS295P1	1:200
OTX2	neural lineages	R&D Systems	AF1979	10 µg/mL
PAX6	neuroepithelium	GeneTex	GTX113241	1:200
Secondary antibodies				
Alexa Flour 594 Goat anti-Mouse IgG2b		Thermo Fisher	A-21145	1:500
Alexa Flour 488 Goat anti-Mouse IgG1		Thermo Fisher	A-21121	1:500
Alexa Flour 488 Goat anti-Mouse IgG		Thermo Fisher	A-11001	1:500
Alexa Flour 568 Goat anti-Rabbit IgG		Thermo Fisher	A-11011	1:500
Alexa Flour 568 Donkey anti-Goat IgG		Thermo Fisher	A-11057	1:500

Table S2. Differentiation and pluripotency marker efficiencies for each differentiation stage of interest. Anticipated efficiencies were obtained through previously published studies which utilized these differentiation protocols [17-20].

Cell Stage	Marker(s)	Anticipated Efficiency	Number of Cells Evaluated	Actual Efficiency
hESCs				
RUES2	OCT4	> 95%	N = 2,263	96.5 %
	SOX2	> 95%	N = 1,405	95.9%
	Nanog	> 95%	N = 1,336	97.1%
Intestinal Lineages				
Day 3: Endoderm	SOX17<br>FOXA2	> 85% double stained	N = 2,045	61.0 %
Day 7: Mid/Hindgut	CDX2	> 90%	N = 1,937	63.8 %
Cardiac Linages				
Day 1: Mesoendoderm	Brachyury	> 90%	N = 1,186	88.7 %
Day 5: Cardiac Mesoderm	Nkx2.5	> 50%	N = 256	98.0 %
Day 8: Cardiac Progenitors	Nkx2.5	> 90%	N = 763	90.0 %
	Isl1	> 90%	N = 843	84.4 %
Day 15: Cardiomyocytes	cTnt	> 80%	N = 2603	80.9 %
Neural Lineages				
Day 5: Intermediate	OTX2	> 50%	N = 372	78.7 %
Day 10: Neuroepithelium	PAX6	> 90%	N = 537	94.6 %
	OTX2	> 90%	N = 198	91.9 %

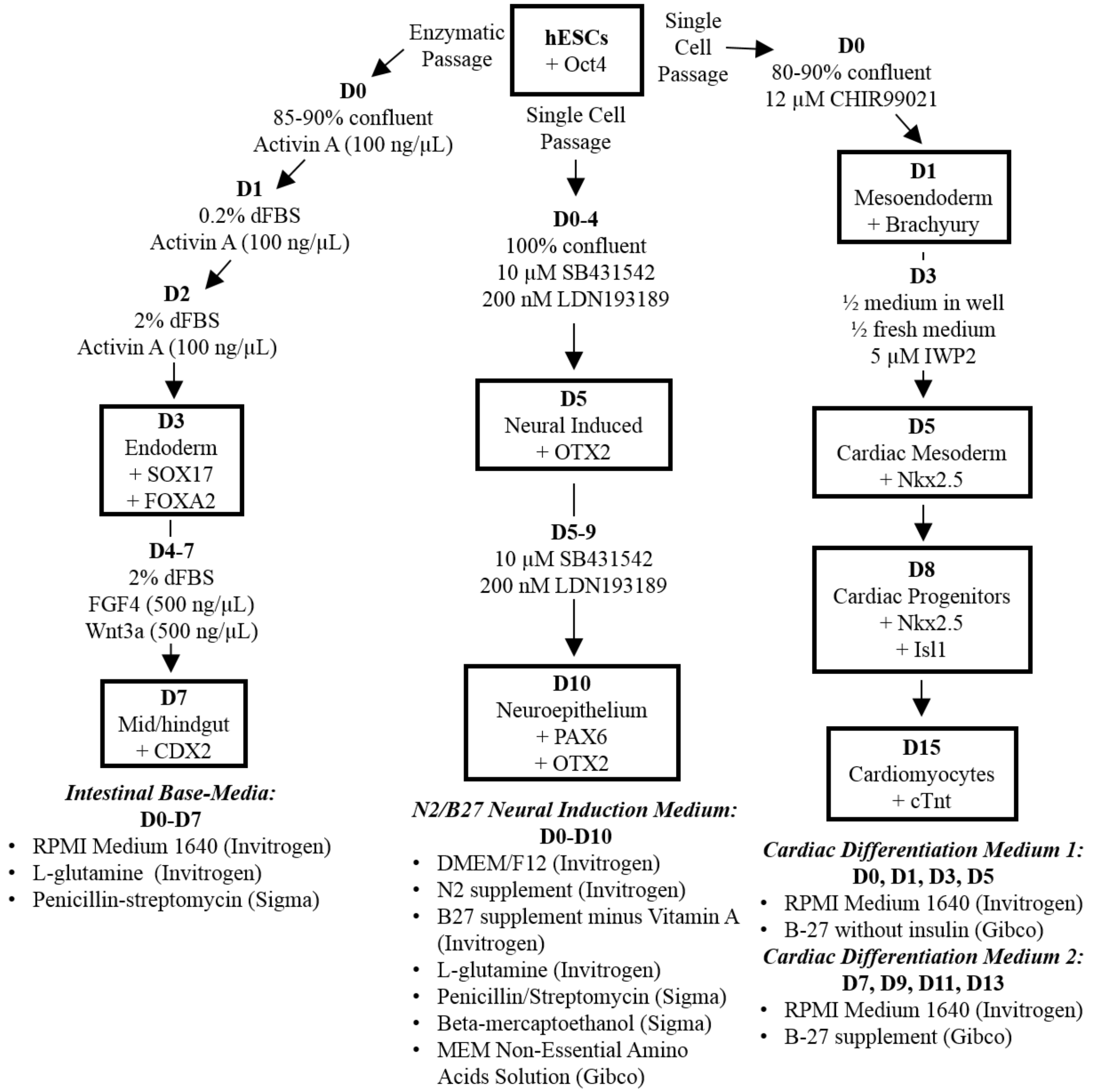

Figure S1. Differentiation of hESCs down three lineages: mid/hindgut on the left, neuroepithelium in the middle and cardiomyocytes on the right. The flow chart shows the type of passaging, level of confluence prior to differentiation, base medium compositions, feeding days and small molecule supplements. The boxes indicate the stages of interest used in this study along with efficiency markers.

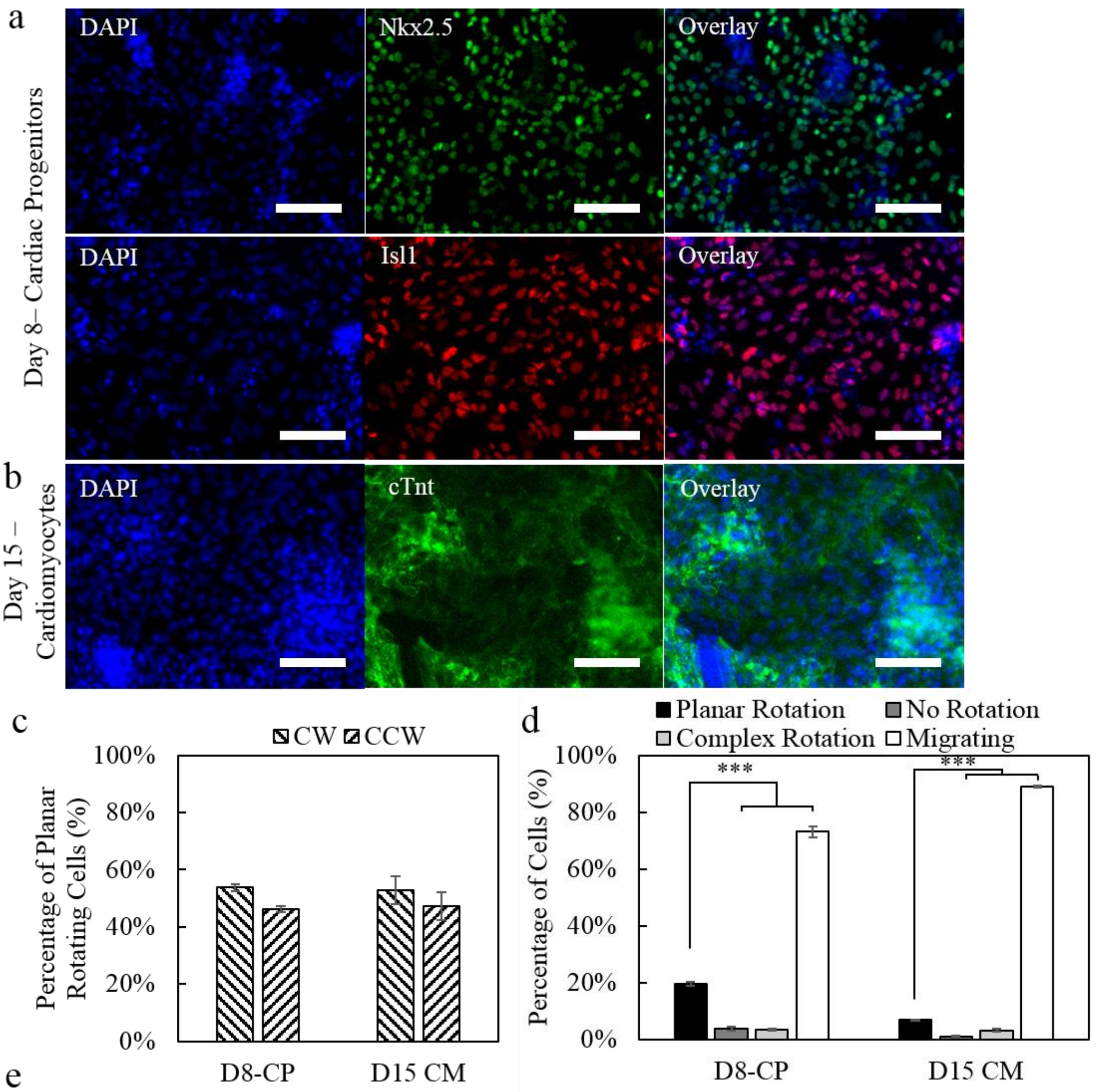

e

Cell Types	N	CW	CCW	p-value	Planar Rotation	No Rotation	Complex Rotation	Migrating	Total	p-value
D8 Cardiac Progenitors	3	30	26	0.344	56	**11**	**9**	**209**	285	2.69E-26
D15 Cardiomyocytes	3	15	13	0.425	28	**4**	**13**	**364**	409	1.40E-80

Figure S2. Later stages of the cardiac lineage show slight CW bias, but are too migratory for accurate evaluation. a) Day 8 cardiac progenitor cells expressing Nkx2.5 and Isl1. b) Day 15 cardiomyocytes expressing cardiac troponin (cTnt). c) Cardiac progenitors (D8-CP) and cardiomyocytes (D15 CM) show slight CW bias which is not statistically significant. d) Both cardiac progenitors and cardiomyocytes exhibit a primarily migratory behavior, significantly decreasing the number of planar rotating cells. e) Quantitative data showing the number of cells in each category along with the number of replicate experiments (N). The p-values indicate comparisons between CW and CCW and between planar rotation and the other three categories. Scale Bars: 100 µm; *** $p < 0.001$

a

Stage of Interest	Marker(s)	Number of Cells Evaluated	Efficiency
**Intestinal Lineages – Staining of 3D Matrigel Bilayer Samples**			
Day 3: Endoderm	SOX17<br>FOXA2	N = 227	85.0 %
Day 7: Mid/Hindgut	CDX2	N = 456	87.2 %

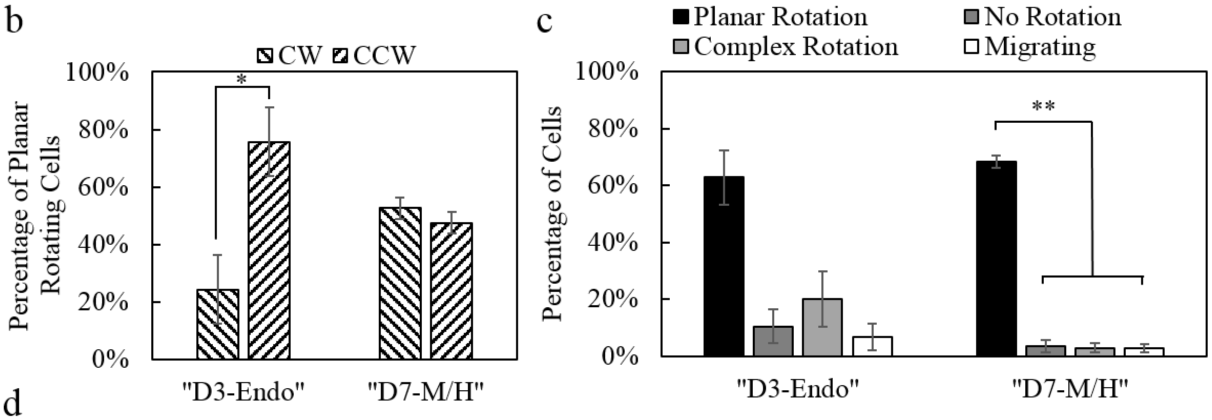

d

Non-Expressing Cell Types	N	CW	CCW	p-value	Planar Rotation	No Rotation	Complex Rotation	Migrating	Total	p-value
"D3 Endoderm"	4	5	**15**	0.021	20	5	7	2	34	0.196
"D7 Mid/Hindgut"	3	20	**19**	0.625	**39**	4	13	2	58	0.006

Figure S3. Non-expressing cells from endoderm and mid/hindgut differentiations. a) Differentiation marker efficiencies for each endoderm and mid/hindgut stages of interest after seeding into the 3D Matrigel bilayer system. b) Cells that did not show dual expressing of endoderm differentiation markers showed a statistically significant CCW rotational bias, but cells that did not express mid/hindgut marker CDX2 showed no chiral bias. c) The non-expressing cells for the mid/hindgut lineage display a significant difference between planar rotating cells and the other categories. d) Quantitative data showing the number of cells in each category along with the number of replicate experiments (N). The p-values indicate comparisons between CW and CCW and between planar rotation and the other three categories. * $p < 0.05$, ** $p < 0.01$

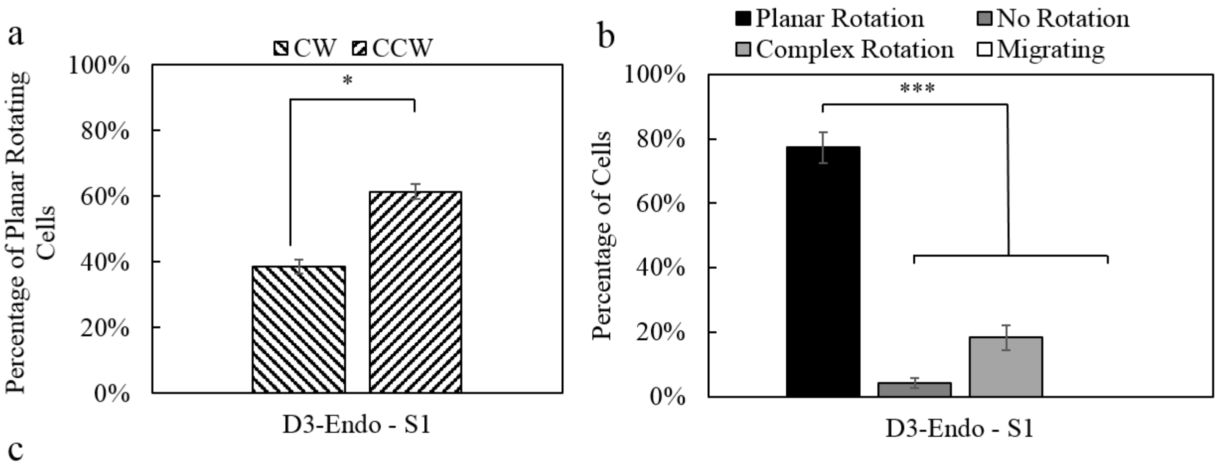

c

Cell Type	N	CW	CCW	p-value	Planar Rotation	No Rotation	Complex Rotation	Migrating	Total	p-value
D3 Endoderm - S1	3	37	**60**	0.012	**97**	6	24	0	127	9.92E-10

Figure S4. Endoderm derived using an alternative differentiation protocol (Pagliuca *Cell* 2014; Rezania *Nat Biotechnol* 2014) also displays a CCW bias. a) Endoderm derived using this protocol (D3-Endo – S1) displays statistically significant CCW rotational bias. b) This endoderm shows significantly more planar rotating cells than other categories. c) Quantitative data showing the number of cells in each category along with the number of replicate experiments (N). The p-values indicate comparisons between CW and CCW and between planar rotation and the other three categories. * $p < 0.05$; *** $p < 0.001$ Reference: Pagliuca FW, *et al.* (2014) Generation of functional human pancreatic beta cells in vitro. *Cell* 159(2):428-439. Rezania A, *et al.* (2014) Reversal of diabetes with insulin-producing cells derived in vitro from human pluripotent stem cells. *Nat Biotechnol* 32(11):1121-1133.

Movie Captions

Video S1. A phase contrast video of a single RUES2 cell rotating counterclockwise over the course of an hour.
Video S2. A tracked version of Video S1, showing a RUES2 cell rotating counterclockwise over the course of an hour.
Video S3. A phase contrast video of a single RUES2 cell rotating clockwise over the course of an hour.
Video S4. A tracked version of Video S3, showing a RUES2 cell rotating clockwise over the course of an hour.
Video S5. A phase contrast video of a single RUES2 cell undergoing out of plane complex rotation over the course of an hour.
Video S6. A tracked version of Video S5, showing a RUES2 cell undergoing out of plane complex rotation over the course of an hour.
Video S7. A phase contrast video of a single RUES2 cell undergoing switching complex rotation over the course of an hour.
Video S8. A tracked version of Video S7, showing a RUES2 cell undergoing switching complex rotation over the course of an hour.
Video S9. A phase contrast video of a single RUES2 cell migrating over the course of an hour.
Video S10. A phase contrast video of a single RUES2 cell undergoing no rotation over the course of an hour.
Video S11. An instructional video detailing how to manually analyze the 3D rotation of cells embedded in the 3D Matrigel bilayer system using ImageJ.
